# Phylogenetic Incongruence in *E. coli* O104: Understanding the Evolutionary Relationships of Emerging Pathogens in the Face of Homologous Recombination

**DOI:** 10.1371/journal.pone.0033971

**Published:** 2012-04-06

**Authors:** Weilong Hao, Vanessa G. Allen, Frances B. Jamieson, Donald E. Low, David C. Alexander

**Affiliations:** 1 Department of Laboratory Medicine and Pathobiology, University of Toronto, Toronto, Ontario, Canada; 2 Mount Sinai Hospital, Toronto, Ontario, Canada; 3 Public Health Laboratories, Public Health Ontario, Toronto, Ontario, Canada; University of Hyderabad, India

## Abstract

*Escherichia coli* O104:H4 was identified as an emerging pathogen during the spring and summer of 2011 and was responsible for a widespread outbreak that resulted in the deaths of 50 people and sickened over 4075. Traditional phenotypic and genotypic assays, such as serotyping, pulsed field gel electrophoresis (PFGE), and multilocus sequence typing (MLST), permit identification and classification of bacterial pathogens, but cannot accurately resolve relationships among genotypically similar but pathotypically different isolates. To understand the evolutionary origins of *E. coli* O104:H4, we sequenced two strains isolated in Ontario, Canada. One was epidemiologically linked to the 2011 outbreak, and the second, unrelated isolate, was obtained in 2010. MLST analysis indicated that both isolates are of the same sequence type (ST678), but whole-genome sequencing revealed differences in chromosomal and plasmid content. Through comprehensive phylogenetic analysis of five O104:H4 ST678 genomes, we identified 167 genes in three gene clusters that have undergone homologous recombination with distantly related *E. coli* strains. These recombination events have resulted in unexpectedly high sequence diversity within the same sequence type. Failure to recognize or adjust for homologous recombination can result in phylogenetic incongruence. Understanding the extent of homologous recombination among different strains of the same sequence type may explain the pathotypic differences between the ON2010 and ON2011 strains and help shed new light on the emergence of this new pathogen.

## Introduction

Identification and classification of infectious agents are key to epidemiological surveillance and public health activities. In practice, however, accurate identification of pathogenic bacteria can be challenging. Genome plasticity [1], [2] confounds classification by creating distinct absence/presence patterns of virulence genes among closely related strains [3] and homologous recombination can generate the illusion of identity between distantly related strains [4], [5]. Pulsed-field gel electrophoresis (PFGE) is a standardized method for comparison of bacterial pathogens. Although, PFGE can identify macroscopic genomic differences among isolates, it provides no specific DNA sequence information and therefore cannot be used for understanding the underlying genetic diversity and evolutionary history of individual strains. Multilocus sequence typing (MLST) has become a routine method for inferring evolutionary relationships [6] and allows distinct strains of similar sequence types to be grouped into clonal complexes [7]. There is a growing body of evidence that a significant amount of genomic diversity, including gene content variation and sequence diversity, exists within a bacterial population in a variety of species [8]–[10]. The existence of genomic and pathotypic variation within sequence types can confound accurate assessment of the evolutionary relationship among apparently similar strains [11]. To achieve better resolution of evolutionary relationships for closely related strains, a large number of universally present genes (or the core genes) have been commonly used in phylogenomic studies [12]–[15].

Homologous recombination has long been recognized in *Escherichia coli* [16], [17], but the frequency of recombination in *E. coli* genomes was generally believed to be less significant than in highly recombinogenic genomes such as *Neisseria* and *Streptococcus* [10], [18]. Recently, substantial phylogenetic incongruence has been observed in the *Escherichia* genus and related genera [19], [20]. Despite the large number of recent phylogenomic studies, the phylogenetic consequence of homologous recombination within the *E. coli* species has not been fully addressed [12], [13], [15], [21]–[23]. It is therefore important to determine the phylogenetic consequence of homologous recombination in phylogenomic analyses of this well studied and model species.

In this study, we sought to address the phylogenetic consequence of homologous recombination in the emerging pathogen, *E. coli* O104:H4. Shiga-toxin-producing *E. coli* (STEC) O104:H4 (sequence type 678) was responsible for a severe outbreak of diarrhea and hemolytic-uremic syndrome (HUS) that originated in Germany in May, 2011 [24]. By July 26^th^, when the outbreak officially ended, the strain had spread throughout Europe and travel-associated cases had been detected in North America [25]. In total, more than 4075 cases were identified, including 908 with HUS and 50 deaths. As part of the international public health response, draft genomes have been obtained for outbreak isolates from several countries [22], [23], [26], [27]. In addition, a historic EHEC ST678 strain causing HUS isolated from 2001 (01-09591) was sequenced [22] and the previously completed genome of strain 55989 [13], an enteroaggregative *E. coli* (EAEC) O104:H4 ST678 strain isolated in Central Africa in the late 1990 s, was identified [28]. Although the rapid generation of sequencing data helped unveil the genetic characteristics of the outbreak strain [22], [23], [26], [27], the evolutionary history and genomic idiosyncrasies of the entire ST678 group remain to be investigated. In this study, we sequenced two additional *E. coli* O104:H4 ST678 genomes: ON2011 was isolated from an adult with epidemiological links to the 2011 outbreak; ON2010 was isolated in 2010 from an infant with a history of travel to the Philippines [25]. Comprehensive phylogenetic analyses of the *E. coli* O104:H4 ST678 group revealed many genes in this group that have undergone homologous recombination and have impacted the evolutionary history of this emerging pathogen. We demonstrate that the presence of recombinant genes in very closely related genomes (e.g., strains in the same sequence type) can seriously mislead phylogenetic interpretation and should be accounted for in order to obtain accurate interpretation on the evolutionary origin of an emerging pathogen.

## Results

### Core genes in *E. coli* and relationship based on the core genes

The five examined *E. coli* O104:H4 ST678 genomes (EHEC ON2011, LB226692, 01-09591; EAEC 55989 and ON2010) shared 4084 genes, but when a total of 58 *Escherichia* and *Shigella* genome were examined the number of core genes decreased to 2085. This is consistent with the previously predicted asymptotic value (about 2,200 genes) in *E. coli* [29]. Our slightly lower value can be explained by the exclusion of duplicated genes and the inclusion of the *Escherichia fergusonii* and *Shigella* genomes in this study. In another recent study [30], which compared 61 *Escherichia* and *Shigella* genomes, including another *Escherichia* species *E. albertii*, a lower core gene estimate of 993 was found. Such a discrepancy is likely due to the larger number of incomplete genomes used, 23 in ref. [30] compared to only 4 in this study. These studies consistently suggest that, with an average genome size of 4.93 Mb (or close to 5000 genes), less than half of the genes are conserved across all *E. coli*/*Shigella* genomes. Such substantial variation in gene content has been previously recognized [31] and shown to be due to the fast turn-over of recently acquired genes during genome evolution [32], [33].

**Figure 1:**
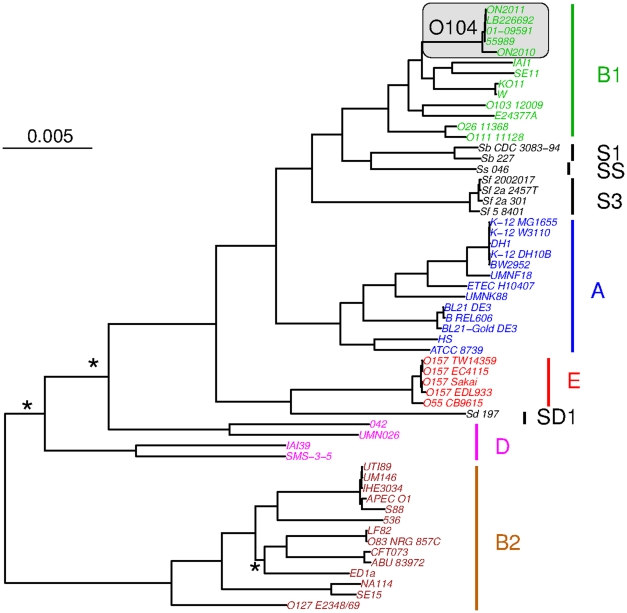
Maximum likelihood phylogenetic tree of the 58 *Escherichia* and *Shigella* strains (57 *E. coli/Shigella* + *E. fergusonii*) as reconstructed from the sequences of 2085 universally present single-copy genes (1962650 characters in total). *E. fergusonii* was chosen to root the tree. Three internal branches that are not well supported (with a bootstrap value <90) are labeled as asterisks. Phylogenetic group membership of the strains is indicated with bars at the right of the figure. The *E. coli* O104 strains are shaded.

**Figure 2:**
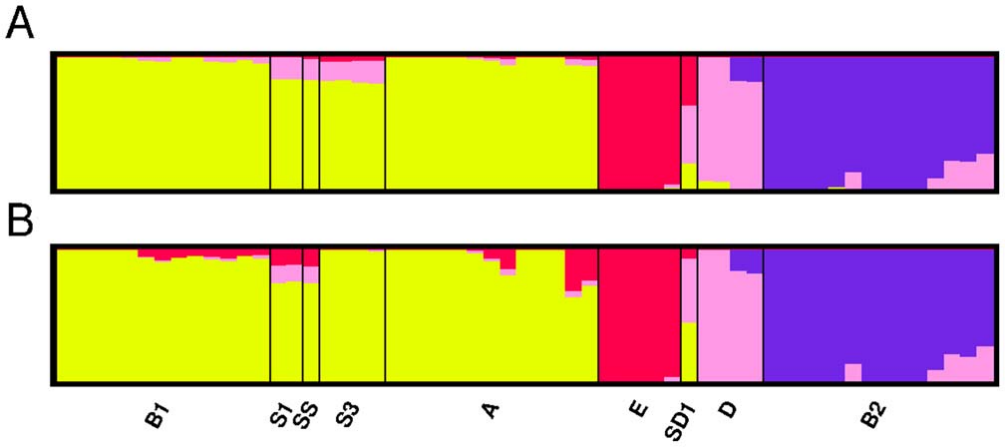
Population clusters of the *Escherichia* and *Shigella* strains. A), all 2085 universally present genes were analyzed. B), universally present recombinant genes were excluded. Proportions of ancestry were inferred using STRUCTURE [35] by assuming four groups (K = 4), and displayed with DISTRUCT [67]. Each column represents one genome, and the genome order is as in Figure 1.

The 2085 core genes were concatenated and a maximum likelihood tree was constructed using the RAxML program [34]. It is shown that all major phylogenetic groups, with the exception of group D, were well supported as monophyletic clades (Figure 1). The major phylogenetic groups were also supported by the population clusters constructed using the STRUCTURE program [35] (Figure 2A). All *E. coli* O104 strains (shaded in Figure 1) formed a monophyletic clade within the B1 group. It is noteworthy that, although all the *E. coli* O104 strains in this study are pathogenic (EHEC for ON2011, LB226692, 01-09591, and EAEC for 55989 and ON2010), they clustered more closely to commensal strains IAI1 [13], SE11 [36], KO11 [37], and W [38], than to other pathogenic strains such as O26 (EHEC), O111 (EHEC), O103 (EHEC) [12], E24377A (ETEC) [30]. The sporadic distribution of the EHEC strains is consistent with previous findings that *E. coli* strains of different phylogenies can independently evolve into EHECs [12]. The phylogeny based on these 2085 genes also showed that, among the *E. coli* O104 strains, ON2011, LB226692, 01-09591, and 55989 were clustered together with very short branches, whereas the ON2010 strain was distinct from other *E. coli* O104 strains by a relatively long branch, e.g., the branch leading to ON2010 is approximately 5.15 times in length as the branch leading to the clade of all other *E. coli* O104 strains (Figure 1).

### Substantially different topologies of the O104 strains using different methodologies

**Figure 3:**
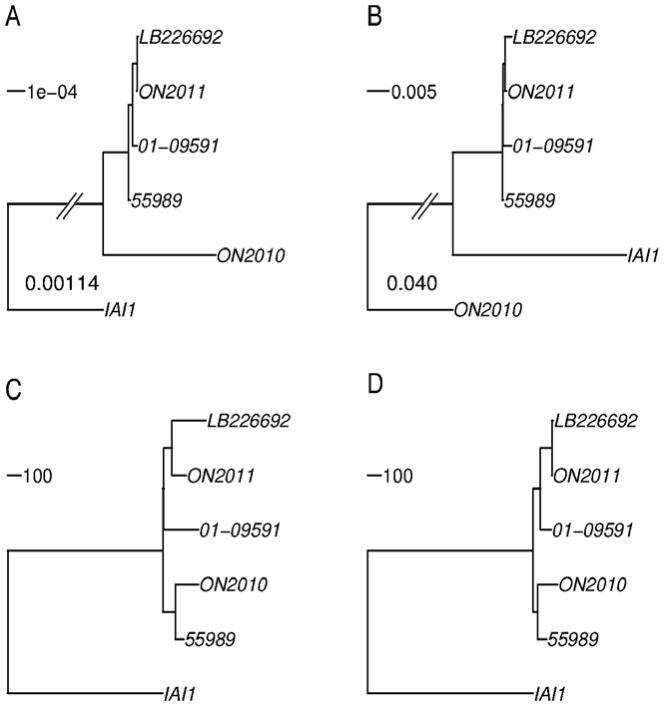
*E. coli* O104:H4 phylogenies constructed based on the 3794 shared genes using different methodologies. A), maximum likelihood tree of the concatenated sequences (3613248 characters). All branches are 100% bootstrap supported. The branch length separating IAI1 from the *E. coli* O104:H4 strains is not to scale and the length is shown. B), feature frequency profiles (FFPs) tree. ON2010 is shown to be distinct from the remaining *E. coli* O104:H4 strains and IAI1. The branch length separating ON2010 from other strains is not to scale and the length is shown. C), neighbor-joining tree based on the number of alleles that differ between any two strains. D), neighbor-joining tree based on the number of alleles that have none-zero DNA distance between any two strains. Unlike in C, small indels, including possible homopolymer sequencing errors, were not considered in D.

Knowing the branch length contrast between the 2010 strain and other *E. coli* O104 strains, we focused our phylogenetic analysis on the *E. coli* O104 strains and used IAI1 as an outgroup (Figure 1). There were 3794 genes shared by the *E. coli* O104 strains and IAI1. First, a maximum likelihood phylogeny was constructed using the concatenated sequences of the 3794 gene alignments (Figure 3A). The topology based on the 3794 genes is essentially identical with the O104 relationship inferred by the 2085 universally present genes in Figure 1. That is, ON2011, LB226692, 01-09591, and 55989 are very closely related, while the ON2010 strain is distinct from others by a long branch. Second, a feature-frequency-profile (FFP) phylogeny was constructed (Figure 3B), since nucleotide composition contains genomic signatures [39] and the word frequency profile can be used for phylogenetic purposes [40]. It is striking that, based on the FFP, the IAI1 strain, which is of serotype O8, ST1128, and shares only two identical MLST loci (out of seven) with ST678, clustered more closely to other O104 strains than the ON2010 strain, even though the ON2010 strain is of both the same serotype (O104) and the same sequence type (ST678) as LB226692, ON2011, 01-09591, and 55989. Figures 3A and 3B revealed that the ON2010 genome contains sequences significantly different from other O104 strains.

**Figure 4:**
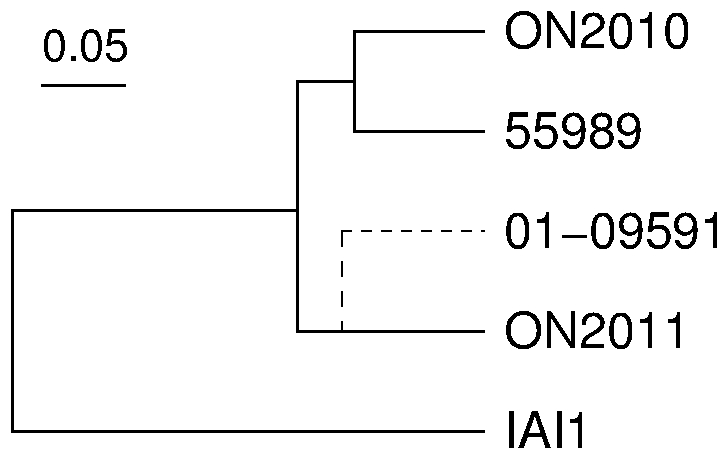
Optical map similarity cluster of the *E. coli* O104:H4 strains. De novo whole genome optical maps from the ON2010 and ON2011 strains were generated using the Argus™ optical mapping system with the *Ncol* restriction enzyme. An in silico genomic map of the 55989 strain was generated in MapSolver™ by applying the *Ncol* restriction pattern. A close relationship between LB226692 and 01-09591 was reported by Mellmann *et al.* 2011 using the same restriction enzyme, and the 01-09591 branch is added as dashed.

Furthermore, the allelic profile, i.e., the number of alleles that differ between any two strains [7] was obtained and treated as pairwise distance among the six genomes (Figure 3C). In contrast to the distant relationship of ON2010 from other *E. coli* O104 strains in Figures 3A and 3B, the ON2010 strain was clustered together with the 55989 strain. In other words, among the 3794 examined genes, the 55989 strain shares more identical genes with the ON2010 strain than with any other strain. All of the examined ON104 genomes except 55989 were generated using next generation sequencing platforms, including technologies (e.g., Roche GS-FLX, Ion Torrent) known to be subject to homopolymer sequencing errors (e.g., indels in homopolymer tracts). To minimize the effect of homopolymer indels, we treated sequences with zero phylogenetic distance measured by DNADIST [41] as identical since indels are not considered in the estimation of phylogenetic distance by most phylogenetic programs including DNADIST. Figure 3D was remarkably similar with Figure 3C, and as expected many branches in Figure 3D, especially external branches, were shorter than their counterparts in Figure 3C. Both Figures 3C and 3D support a close relationship between 55989 and ON2010, which is substantially different from the relationship observed in either Figure 3A or Figure 3B. To make better sense of these sequence-based topologies, we constructed a phylogeny using optical mapping by comparing the profile of restriction sites in each genome [22], [25]. The optical mapping phylogeny also revealed a close relationship between 55989 and ON2010 (Figure 4).

### Conserved genome synteny but varied sequence diversity along the ON2010 genome

**Figure 5:**
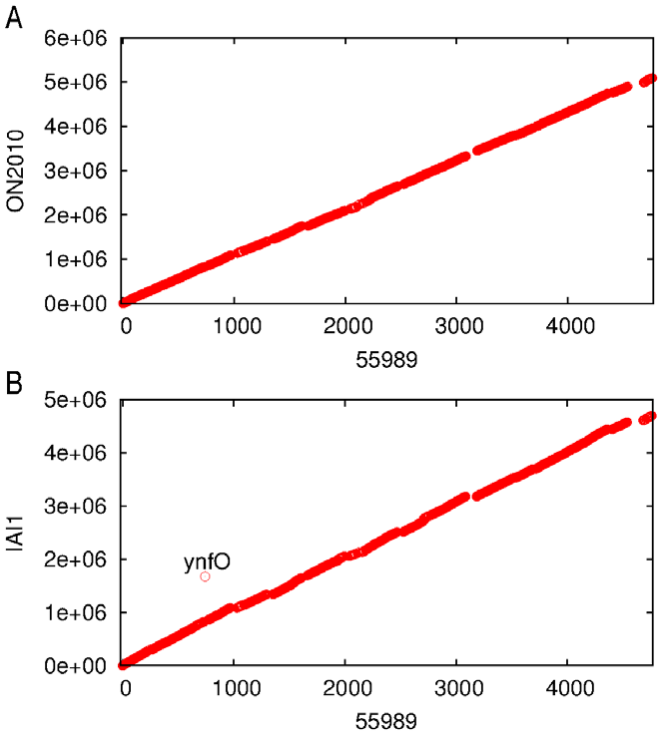
Genome synteny. (A) 55989 vs. ON2010; (B) 55989 vs. IAI1. Homologous matches are taken to have an expected value <10^−20^ for all the 3792 genes shared by IAI1, 55989, 01-09591, ON2010 and ON2011 in a BLASTN search. The x-axis shows the order of genes on the 55989 chromosome. The y-axis shows the nucleotide coordinates of the subject genome.

Genome synteny was plotted using the 3794 genes shared by the *E. coli* O104 strains and the IAI1 strain (Figure 5). Consistent with the optical mapping data, the scaffold of the ON2010 genome and the complete 55989 genome showed conserved genome synteny and no large scale genome rearrangement was observed. Similarly, the 55989 genome and the IAI1 genome also showed conserved genome synteny with only one gene (a Qin prophage gene *ynfO*) under gene translocation (Figure 5). This is consistent with previous work indicating that compared to many other species such as *Neisseria meningitidis*, *Streptococcus pneumoniae* and *Wolbachia*, *E.coli* genomes are relatively stable [42].

**Figure 6:**
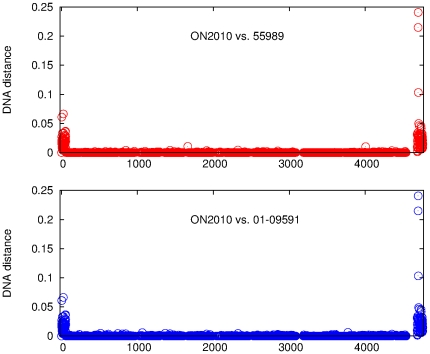
DNA distance between ON2010 vs. 55989 and between ON2010 vs. 01-09591. The data are plotted as of the gene order on the 55989 chromosome.

Nucleotide divergence was examined by measuring the DNA distance of each individual gene in ON2010 against its homolog in 55989 or 01-09591 (Figure 6). It is striking that, when comparing ON2010 vs. 55989, and ON2010 vs. 01-09591, genes at the beginning and the end of the genome (125 genes in total) showed substantially elevated DNA distance compared to the rest of the genome. The DNA sequences of the 125 genes from ON2010 are available at https://sites.google.com/site/haowlab/. Homologs of these 125 genes in ON2011 were also examined, the comparision between ON2010 vs. ON2011 yielded essentially identical results as that between ON2010 vs. 01-09591 (data not shown). Since the *E. coli* O104 strains have conserved genome synteny and the genomes are circular, the most parsimony explanation would be that these 125 genes have been horizontally acquired into the ON2010 genome by a single homologous recombination event.

**Figure 7:**
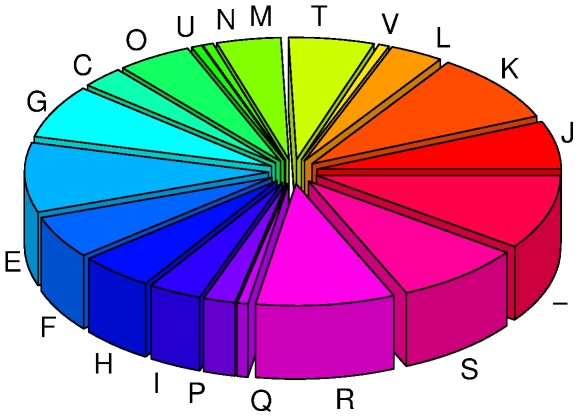
COG functional categories of the 125 genes involved in ON2010-specific recombination. The functional categories are information storage and processing, including COG categories J, K, L, and B; cellular processes and signaling, including V, T, M, N, U, and O; metabolism, including C, G, E, F, H, I, P, and Q; poorly characterized including R and S; and ‘-’ refers to not in COG.

It is worth noting that the sequence length involved in these 125 genes is much longer than the unit of a biosynthesis cluster, which generally contains less than 10 genes. It is therefore unlikely that the retention of these 125 genes in ON2010 is directly associated with directional selection for a single functional unit, such as a biosynthesis pathway. In contrast, these 125 genes were found in 20 different functional categories according to the COG (Clusters of Orthologous Groups of proteins) classifications [43] (Figure 7). One type of large scale gene movement in bacterial genomes is in the form of superintegron, which generally contains a cluster of gene cassettes [44], [45]. The superintegron in *Vibrio cholera*, contains at least 215 ORFs [46]. However, there is no direct evidence that the 125 recombinant genes in ON2010 were associated with integron recombination. All integrons contain characteristic genes cassettes and encode an *IntI* integrase [47]. These features were not observed. Although two partial matches to *intI* were found on the ON2010 chromosome, they were located more than 1.8Mb away from the recombinant gene region (data not shown). We also investigated any direct evidence that the recombinant genes were associated with repetitive sequences, but no repeat sequences were detected in the 10 Kb flanking the recombinant region (from the *gntP* gene to the *setA* gene). Furthermore, a comparison against the annotated prophage regions in 55989 revealed that these 125 genes are not associated with prophages (Figure S1). Given the fact that *E. coli* O104 genomes all exhibit extensive synteny (Figure 5) and recombination plays a crucial role in the speciation of enterobacteria [19], [48], it is very likely that the 125 recombinant genes were acquired via direct chromosomal homologous recombination.

### Recombinant genes in ON2010 resulted in the incongruence of phylogenies constructed by different methodologies

**Figure 8:**
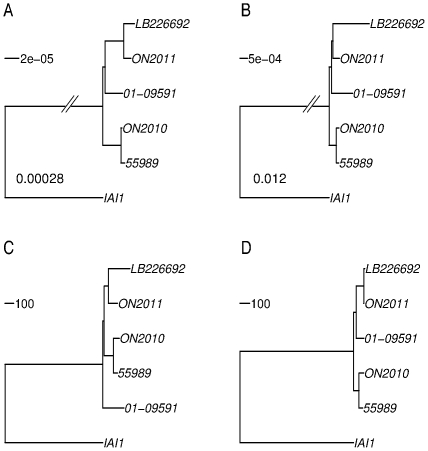
*E. coli* O104:H4 phylogenies constructed after the removal of the 125 gene involved in recombination in ON2010. A), maximum likelihood tree of the concatenated sequences of 3669 genes (3487410 characters). All branches are 100% bootstrap supported. B), feature frequency profiles (FFPs) tree. C), neighbor-joining tree based on the number of alleles that differ between any two strains. D), neighbor-joining tree based on the number of alleles that have none-zero DNA distance between any two strains. Unlike in C, small indels, including homopolymer sequencing errors, were not considered in D.

Given that foreign genes could introduce conflicting phylogenetic signals, one should expect that the removal of the recombinant genes would reduce the substantial incongruence observed in Figure 3. In fact, after the removal of these 125 genes, remarkably similar phylogenetic relationships were obtained by different methodologies (Figure 8). Figures 8A, 8B, and 8D revealed an essentially identical topology by placing the two 2011 German outbreak strains together with the 2001 EHEC strain 01-09591, and ON2010 together with 55989. In Figure 8C, the two 2011 German outbreak strains appear more closely related to the ON2010-55989 clade than 01-09591. The difference is likely due to some homopolymer sequencing errors as the phylogeny based on the zero-distance (Figure 8D) is topologically identical with Figures 8A and 8B. Along the same line, the observed elongated branches leading to LB226692 and 01-09591 in Figure 8B could also be due to homopolymer sequencing errors. The removal of the recombinant genes resulted in much greater contrast in sequence-based phylogenies (Figures 3A vs. 8A, and 3B vs. 8B) than in allele-based phylogenies (Figures 3C vs. 8C, and 3D vs. 8D). The results suggest that although sequence-based phylogeny construction is generally more sophisticated than allelic profiling, in the case of a small number of foreign genes contributing to a large majority of the nucleotide changes among closely related strains, sequence-based phylogenies can be more seriously misled than allele-based phylogenies.

Even though the existence of recombination in *E. coli* has long been recognized [16], [17], the consequence of recombination has not been well appreciated in recent phylogenomic studies. Routinely, the concatenated sequences of a large number of commonly shared genes were used for phylogenomic analysis [13], [15], [22], [23], [49], but most of these studies except [15] did not screen for or exclude genes involved in intragenic recombination or horizontal gene transfer. In this current study, phylogenetic incongruence was observed when 70 (of the 125 identified) recombinant genes were included in the set of 2085 universally present genes (Figure 1). Notably, a similar proportion of recombinant genes (36 in a core set of 1144) were present in a previous phylogeny construction of the O104 strains [22]. Comparison of *E.coli/Shigella* phylogenies generated before (Figure 1) and after the removal of recombinant genes (Figure S2) from the universally present gene set indicates that, despite the absence of topological changes in the tree as a whole, significant changes occurred among the *E. coli* O104 strains. That is, when the examined sequences are reasonably divergent, the inclusion of a small number of foreign genes introduced little phylogenetic incongruence. Similary, the population clusters constructed by STRUCTURE [35] remained remarkably similar after the removal of the 70 recombinant genes (Figure 2). It is also notable that mixed ancestry patterns were observed in several genomes (Figure 2), which are due to relatively ancient recombination events. Similar results were found among the major modern *E. coli* groups by Wirth et al. [50]. Given the existence of recombination, one must be extremely cautious on how to interpret the well supported (e.g., by high bootstrap values) phylogenies. The fact is that well supported phylogenomic trees of concatenated sequences do not necessarily suggest clonal relationships of the examined strains. This is consistent with previous, independent analyses showing that chimeric sequences of 25–30% foreign and 70–75% native origins could still resemble native sequences in phylogenetic analysis [51], [52]. However, as observed here in the *E. coli* O104 genomes, even a relatively small number (72/2085; 3.4%) of foreign genes could severely alter the phylogeny if the examined sequences are virtually identical.

### Genes in ON2010 involved in recombination with different distantly related strains

We then identified the nearest phylogenetic neighbors of these 125 recombinant genes. Surprisingly, the nearest neighbors of these 125 genes were not clustered in a single phylogenetic group. While a large portion of them (56 or 44.8%) were in the D group, more than half of the nearest neighbors were spread into six phylogenetic groups, there were 27 in the B1 group (21.6%), 24 in the B2 group (19.2%), and the remaining 18 in two other *E. coli* groups and two *Shigella* species (Table 1). This suggests that the 125 genes in the ON2010 genome are of different origins. One possibility is that these 125 genes resulted from multiple recombination events with strains from different phylogenetic groups. It is also possible that extensive recombination with a broad spectrum of strains has taken place in one existing genome (possibly from the D group), and this highly mosaic genome then recombined with the precursor to the ON2010 genome. Given the close linkage of these recombinant genes in 2011 and the high similarity of non-recombinant genes among the *E. coli* O104 strains, we tend to favor the latter scenario.

**Table 1:** Distribution of the nearest neighbors of the 125 putative recombinant genes in the ON2010 strain.

Phylogenetic group	Number of nearest neighbors
B1	27
S1	3
SS	2
A	10
E	3
D	56
B2	24

When identical distance was observed in different groups for a gene, the nearest neighbor was assigned to the group most closely related to the B1 group.

**Figure 9:**
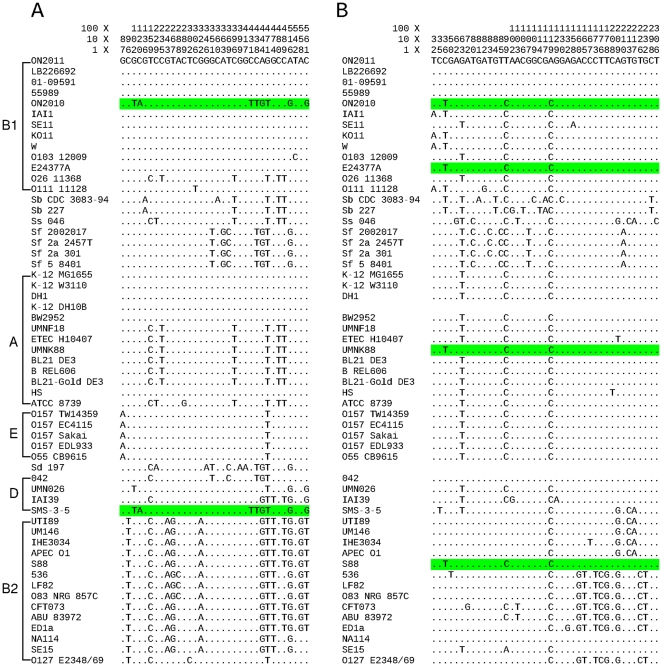
Sequence alignments of *yaaH* (A) and *EC55989_4986* (B). Only informative sites are shown with coordinates at the top. Sequences that are identical with the ON2010 sequence are highlighted in light green.

**Figure 10:**
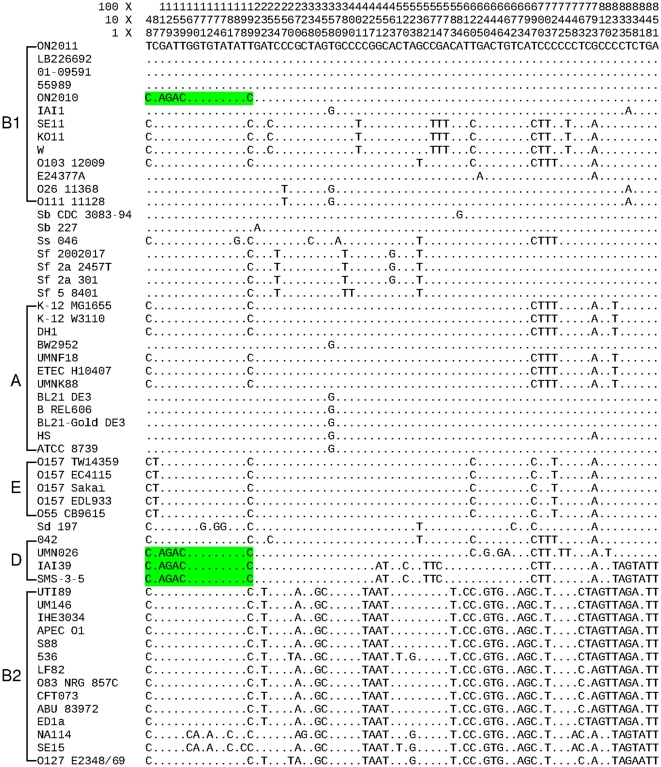
Sequence alignments of the *araC* gene. Regions that are identical with the ON2010 sequence are highlighted in light green.

Figure 9 shows two genes that exhibit sequence variations and strain distributions indicative of recombination. The *yaaH* gene in ON2010 differs by eight nucleotides from all other O104 genomes, but is identical with the allele in SMS-3-5, a group D strain. For gene *EC55989_4986*, the ON2010 allele differs by three nucleotides from other *E. coli* O104 genomes. However, the ON2010 sequence is identical with a B1 strain, E24377A, an A strain UMNK88, and a B2 strain S88, while the sequences in the other O104 genomes are identical with alleles in a D strain (042) and a B2 strain (NA114). It is clear that strains in the same phylogenetic group do not necessarily share the same sequences, and strains containing the same sequence of one gene might not share the same sequence of another gene. Furthermore, intragenic recombination was observed in the ON2010 genome. The *araC* gene in ON2010 differs by six nucleotides from other *E. coli* O104 genomes, and the six nucleotide changes were concentrated at the 5′ end of the gene (Figure 10). Although the entire *araC* sequence in ON2010 is not identical with any other sequence, the 5′ end of the sequence was found to be identical with three group D strains UMN026, IAI39, and SMS-3-5. That is, that the 5′ end of the *araC* gene in ON2010 resulted from intragenic recombination from a strain similar to UMN026, IAI39 or SMS-3-5 while the rest of the *araC* gene in ON2010 remained O104-like. The presence of intragenic recombination was also evident by different recombination detection programs (P = 3.59×10^−04^ in RDP, P = 3.46×10^−06^ in Phi, P = 5.29×10^−07^ in OnePop).

### Evidence of recombination in other *E. coli* O104 genomes

**Figure 11:**
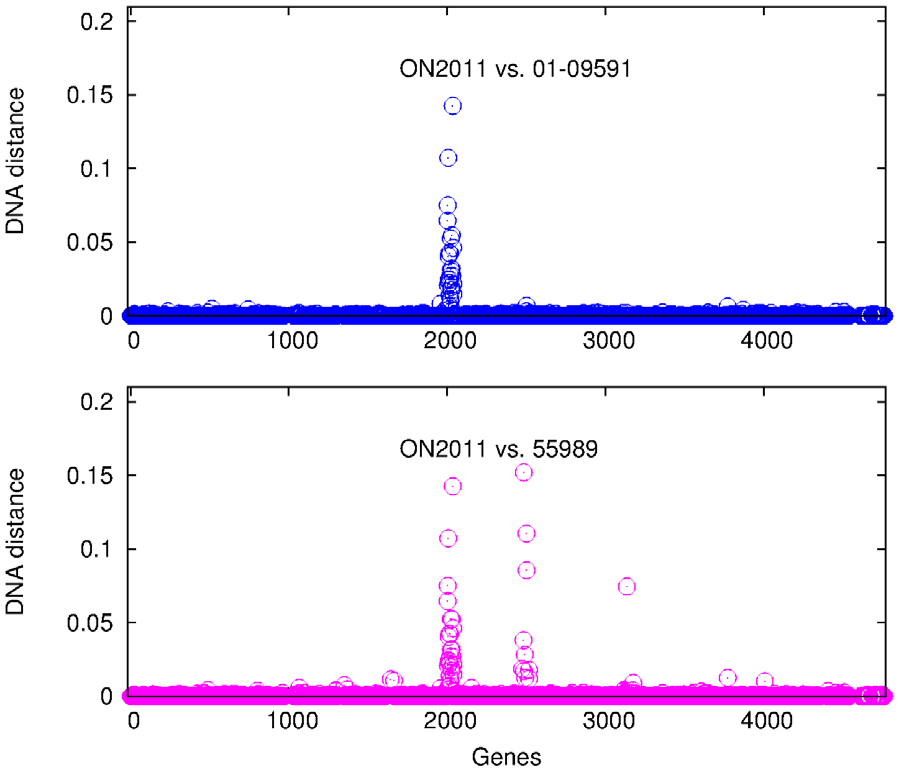
DNA distance between ON2011 vs. 55989 and between ON2011 and 01-09591. The data are plotted as of the gene order on the 55989 chromosome.

Although our initial analyses focused on strain ON2010, evidence of homologous recombination was also observed in other *E. coli* O104 genomes. Comparison of ON2011 with 55989 and 01-09591 was conducted using the same set of genes as in Figure 6 (i.e., the 3794 genes present in the *E. coli* O104 strains and the IAI1 strain). No substantially elevated divergence along the genomes was observed (data not shown). However, when all 4084 *E. coli* O104 genes were examined, two regions of substantially elevated divergence were observed (Figure 11). One region was observed in both the ON2011-01-09591 pair and the ON2011-55989 pair, and they are the genes that have been changed specifically in the ON2011 genome. The second region of genes of substantially elevated divergence was only observed in the ON2011-55989 pair. Given the close relationship between ON2011-01-09591 and between ON2010-55989 (Figures 4 and 8), the observed divergence between ON2011 and 55989 could best be explained by recombination events specific to the ON2011-01-09591 clade or to the ON2010-55989 clade.

We then sought to determine, for the genes in the second region, whether recombination has taken place in the ON2011-01-09591 clade or in the ON2010-55989 clade. The recombinant genes in this region were absent from the IAI1 genome, but they were found in the W strain. The DNA distance was estimated for each gene in this region from ON2011, 01-09591, ON2010, or 55989 against the W strain (Figure S3). Strikingly, a closer relationship to the W strain was observed in some ON2011 (and 01-09591) genes and in some 55989 (and ON2010) genes. In other words, these clustered genes in either the ON2011-01-09591 clade or the ON2010-55989 clade are most likely of different origins. Unlike the 125 recombinant genes specific to ON2010 (Figure 6), the 42 genes in the two recombinant regions in Figure 11 were associated with prophages, Such recombination in large genome segments are very unlikely to be detected by recombination detection programs, since recombination detection programs were designed to detect intragenic recombination and many of them, including RDP, Phi, and OnePop, do not incorporate existing phylogenetic relationships in the analysis. Some methods (e.g., the SH test and the AU test) were designed to detect phylogeney incongruence, however, the successful detection of foreign genes from a large amount of phylogenomic data can be challenging due to the demands of fast computation using hierarchical comparison [53]. Furthermore, it has been shown that, for the same parental and recombinant sequences, the inclusion of many distantly related outgroup sequences could lead to a poorer performance of recombination detection using phylogenetic methods [54]. Therefore, plotting nucleotide divergence (DNA distance) along the genome provides a powerful visual assessment of recombinant genes from nearly identical genes.

### Evidence of recombination in the *E. coli* O104 plasmids

Knowing the mosaic evolution of the *E. coli* O104 chromosomes, investigation was conducted on the evolutionary history of the homologous pAA plasmid genomes. The 55989 strain contains a single plasmid encoding aggregative adherence fimbria (AAF/III) [14], the same type of plasmid was found in 01-09591 [22], and the pAA plasmid in the Germany outbreak strain was shown to be a rare type AAF/I instead of the more common AAF/III [27] (also shown in Figures S4 and S5). The pAA plasmid in the ON2010 strain from this study was remarkably similar with the 55989 plasmid and they differ only by 13 nucleotides (Table 2). The 01-09591 strain contains three plasmids, the pAA plasmid is the second biggest plasmid (GenBank: AFPS01000102) [22]. It is slightly longer than the 55989 plasmid (72573 nt *vs*. 72482 nt) but the two are identical at 72410 nucleotides. The remarkable similarity of pAA plasmids from 55989 and ON2010 is consistent with the close relationship between 55989 and ON2010 supported by chromosomal sequences (Figures 4 and 8). The pAA plasmid corresponds to the second largest plasmid in the German outbreak strain [27], but the outbreak strain exhibits distinct regions of gene gain/loss (Figures S4 and S5).

**Table 2:** Differences between the plasmids in the ON2010 and 55989 strains.

Locus	55989			ON2010		
	coordinate	nt	aa	coordinate	nt	aa
intergenic	4644	A	-	4644	G	-
pEC55989_0015	10225	C	G	10225	A	V
intergenic	23233-23256	(GTAGCA)_4X_	-	23233-23250	(GTAGCA)_3X_	-
pEC55989_0047	31535	C	G	31529	A	V
intergenic	52282	-	-	52276	A	-
pEC55989_0090	59453	A	S	59448	T	C
intergenic	60217	A	-	60212	G	-
pEC55989_0095	64740	T	K	64735	C	E

Complete pAA sequences are available for two other strains, 042 and p086A. Figure S5 shows that homologous sequences could have variable sequence similarity among the genome in addition to the distinct presence/absence pattern for several regions. Analysis of DNA distance among the homologous sequences revealed that some regions in the ON2011 pAA plasmid are highly similar with other *E. coli* O104 plasmids, while some other regions in the ON2011 pAA plasmid are highly similar with 042 pAA or pO86A (Figure S6). Note that strain 042 belongs to the D group, which is considered the primary donor for the ON2010-specific recombinant genes (Table 1). It is possible that 1) the ON2011 strain might have acquired a plasmid from a group D strain and, 2) after that acquisition, the new pAA plasmid underwent recombination with the ancestral O104 pAA plasmid.

## Discussion

Prior to the 2011 outbreak, *E. coli* O104:H4 (ST678) had only been associated with sporadic illness [25]; [55]. The presence of virulence loci, including a prophage encoding Shiga toxin 2, may explain the increased pathogenity of the 2011 outbreak strain [23], [24] and highlights the way in which the horizontal acquisition of new genes facilitates the emergence of new pathogens. In this study, we have examined the genomes of additional *E.coli* O104:H4 isolates and shown evidence that recombination is an important force driving sequence divergence and should be considered a significant factor in shaping *E. coli* populations. This analysis emphasizes that failure to account for sequence divergence can lead to phylogenetic incongruence among closely related strains. In the particular case that a small number of genes are of foreign origin, the phylogenetic interpretation involved in the recombinant strain and its close relatives can be more seriously misled when using sequence based methods than allelic profiling. For example, in our initial analysis, the close evolutionary relationship between the ON2010 and 55989 strains was confounded by the presence of a relatively small set of recombinant genes. Assuming a genome size of 5 Mb and an estimated mutation rate at 4.5×10^−9^ per year [56], the expected genome wide change in *E.coli* is only 0.0225 mutations per genome per year. In contrast, we found that, in ON2010, homologous recombination altered 3.4% of the core gene set. In other words, single point mutations *per se* in bacterial pathogens are unlikely to play as significant a role as horizontal gene transfer. Despite the utility of traditional methods for bacterial identification and classification, higher resolution methods are necessary to understand the evolution and emergence of new pathogens. To be effective, pathogen surveillance should include methods that permit the detection of gene gain or loss and homologous recombination.

## Methods

Genome sequences for the two *E. coli* O104:H4 isolates from Ontario, Canada, namely ON2010 and ON2011, were generated via Roche GS-FLX pyrosequencing using paired-libraries and Titanium chemistry [25]. Draft genomes were assembled using the gsAssembler (Roche) and CAP3 [57] assemblers, and further edited and visualized using the Phred/Phrap/Consed software package [58]. This Whole Genome Shotgun project has been deposited at GenBank under the accession AHZE00000000, AHZF00000000. The version described in this paper is the first version, AHZE01000000, AHZF01000000. Dotplot was generated for plasmid genomes using Dotter [59]. Genomic content variation in plasmid genomes was illustrated using BLAST Ring Image Generator (BRIG) [60]. Genome sequences for additional *E. coli* O104:H4 strains (n = 3), non-O104 *E. coli* strains (n = 44), *E. fergusonii* and *Shigella* strains (n = 8) were obtained from the GenBank database.

Homologs were identified using BLASTN [61] with an E-value <10^−20^ and match length >85%. To identify orthologs, we required genes present as single-copy per genome. Orthologous sequences were aligned using MUSCLE [62]. Phylogenetic trees were constructed using a maximum likelihood method via the RAxML program [34] under a GTR+Γ+I substitution model. DNA distance among aligned sequences was measured using DNADIST of the PHYLIP package [41] version 3.6. The taxon with the shortest phylogenetic distance was identified as the nearest neighbor [63]. Intragenic homologous recombination was detected using Phi [64], RDP [65], and OnePop [66]. Proportions of ancestry were inferred using STRUCTURE [35] by assuming four groups (K = 4) on the 2085 core genes, the plots were displayed with DISTRUCT [67].

De novo whole genome optical maps from the ON2010 and ON2011 strains were generated using the Argus™ optical mapping system with the *Ncol* restriction enzyme [22] [25]. An *in silico* genomic map of the 55989 strain was generated in MapSolver™ by applying the *Ncol* restriction pattern. Optical maps and *in silico* maps were compared using the default MapSolver™ parameters and clustered using the UPGMA method based on the resulting pairwise distance metrics. Furthermore, evolutionary relationships were constructed using two additional methodologies, 1) an alignment free comparison tool, the FFP (Feature frequency profile) package [40], 2) the number of alleles that differ between any two strains was treated as pairwise distance as described in BIGSdb [7]. Both methodologies have recently been used for phylogenetic analysis in *E. coli* [22], [68].

## Supporting Information

Figure S1 **Recombinant genes and regions associated with prophages.** Clade-specific recombination (either in ON2010-55989, or in ON2011-01-09591, see text for detailed discussion) is colored in magenta.
(TIF)

Figure S2 **Maximum likelihood phylogenetic tree of the 57** ***Escherichia coli*** **and** ***Shigella*** **strains as reconstructed from the sequences of 2013 universally present single-copy genes (1890550 characters in total, after the removal of 70 recombinant genes).** *E. fergusonii* was chosen to root the tree. Three internal branches that are not well supported (with a bootstrap value <90) are labeled as asterisks. The phylogeny shows identical topological relationships as in Figure 2 for all but the O104 strains.
(TIF)

Figure S3 **DNA distance of the second recombinant region in** **Figure 11** **.** DNA distance was measured against the W strain.
(TIF)

Figure S4 **Dot plot of related pAA plasmid genomes.** The likely relationship of corresponding chromosomes is shown on the top, and currently there is no complete chromosome sequence associated with pO86A.
(TIF)

Figure S5 **BRIG analysis of pAA plasmid genomes using the pAA plasmid in ON2011 as the query sequence.**
(TIF)

Figure S6 **DNA distance among related plasmid genomes.** Non-overlapped 500-nucleotide fragments from the second plasmid in ON2011 were used as query sequences. Each data point is based on the DNA distance between the query and subject sequence alignment.
(TIF)
