# Supplementary figures and images for: Phylogenetic Incongruence in E. coli O104: Understanding the Evolutionary Relationships of Emerging Pathogens in the Face of Homologous Recombination
Source: PLoS One. 2012 Apr 6;7(4):e33971. doi: 10.1371/journal.pone.0033971 (PMC3320906; doi:10.1371/journal.pone.0033971)

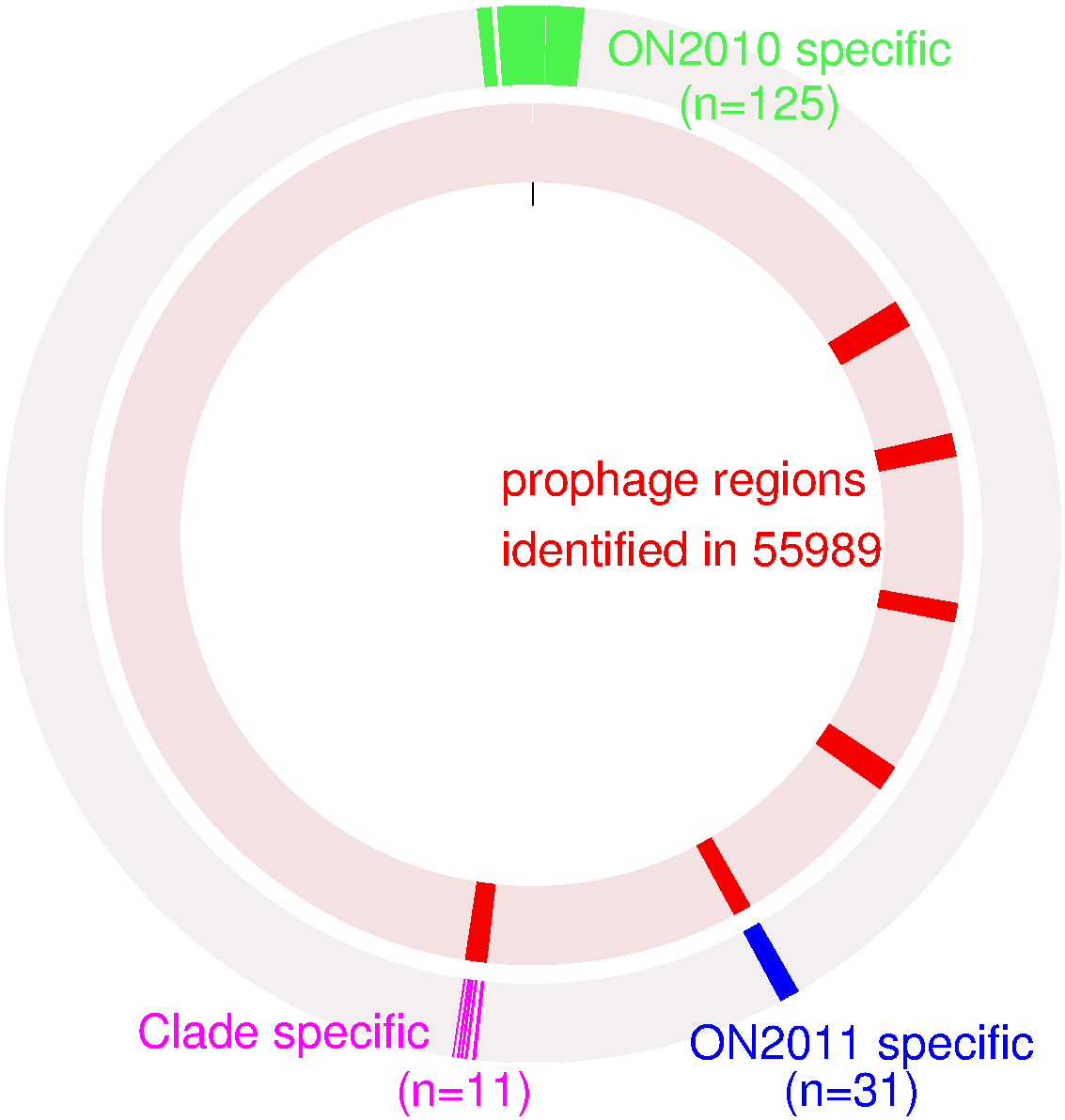

Supplement: Figure S1 — Recombinant genes and regions associated with prophages. Clade-specific recombination (either in ON2010-55989, or in ON2011-01-09591, see text for detailed discussion) is colored in magenta. (TIF) [file pone.0033971.s001.tif]

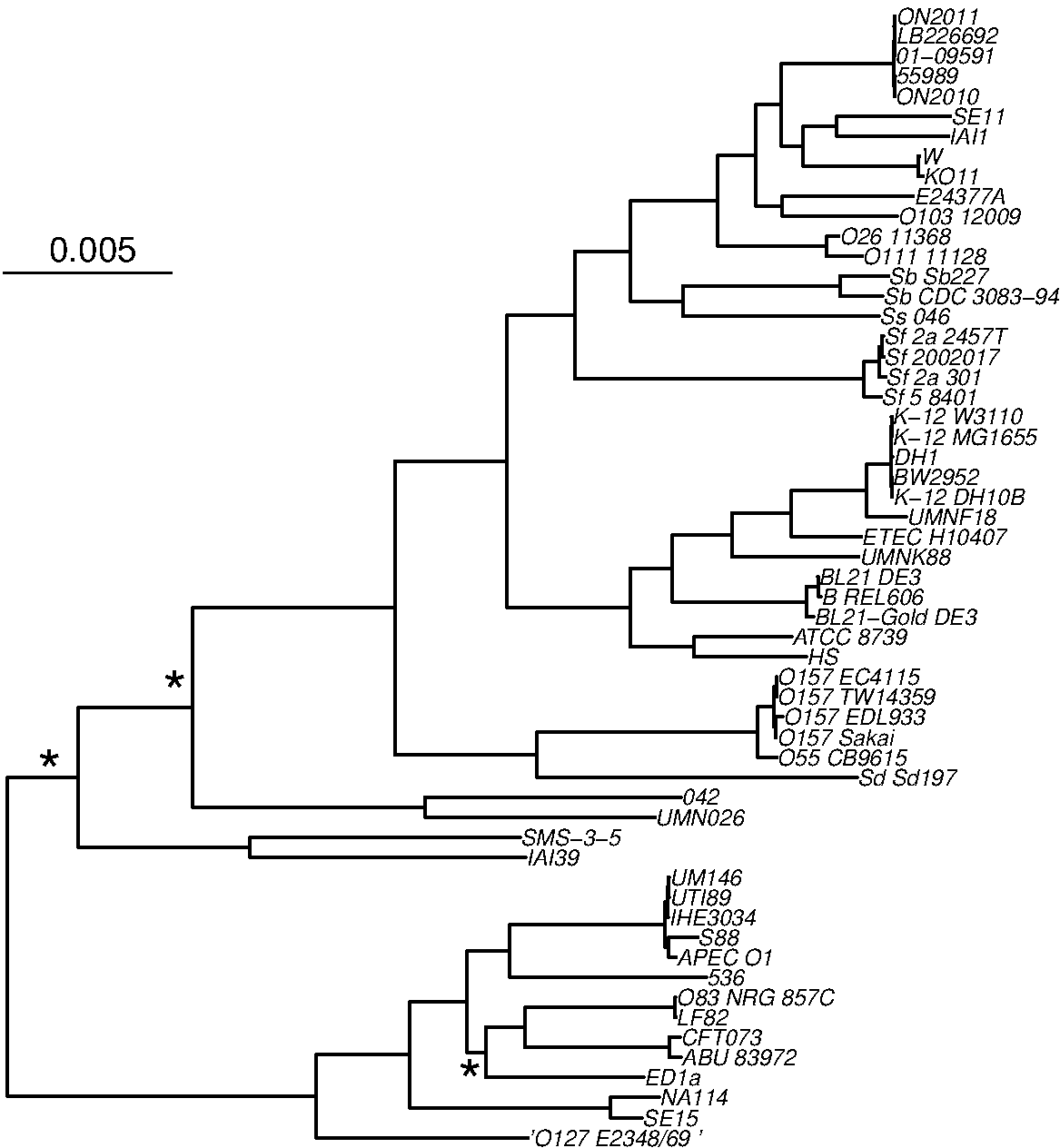

Supplement: Figure S2 — Maximum likelihood phylogenetic tree of the 57 Escherichia coli and Shigella strains as reconstructed from the sequences of 2013 universally present single-copy genes (1890550 characters in total, after the removal of 70 recombinant genes). E. fergusonii was chosen to root the tree. Three internal branches that are not well supported (with a bootstrap value <90) are labeled as asterisks. The phylogeny shows identical topological relationships as in Figure 2 for all but the O104 strains. (TIF) [file pone.0033971.s002.tif]

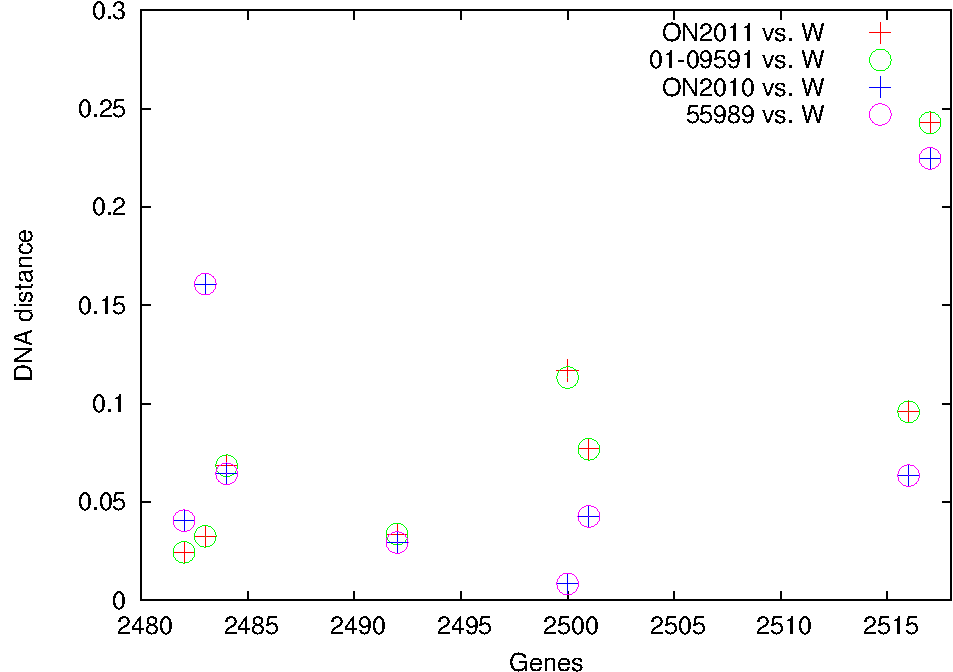

Supplement: Figure S3 — DNA distance of the second recombinant region in Figure 11 . DNA distance was measured against the W strain. (TIF) [file pone.0033971.s003.tif]

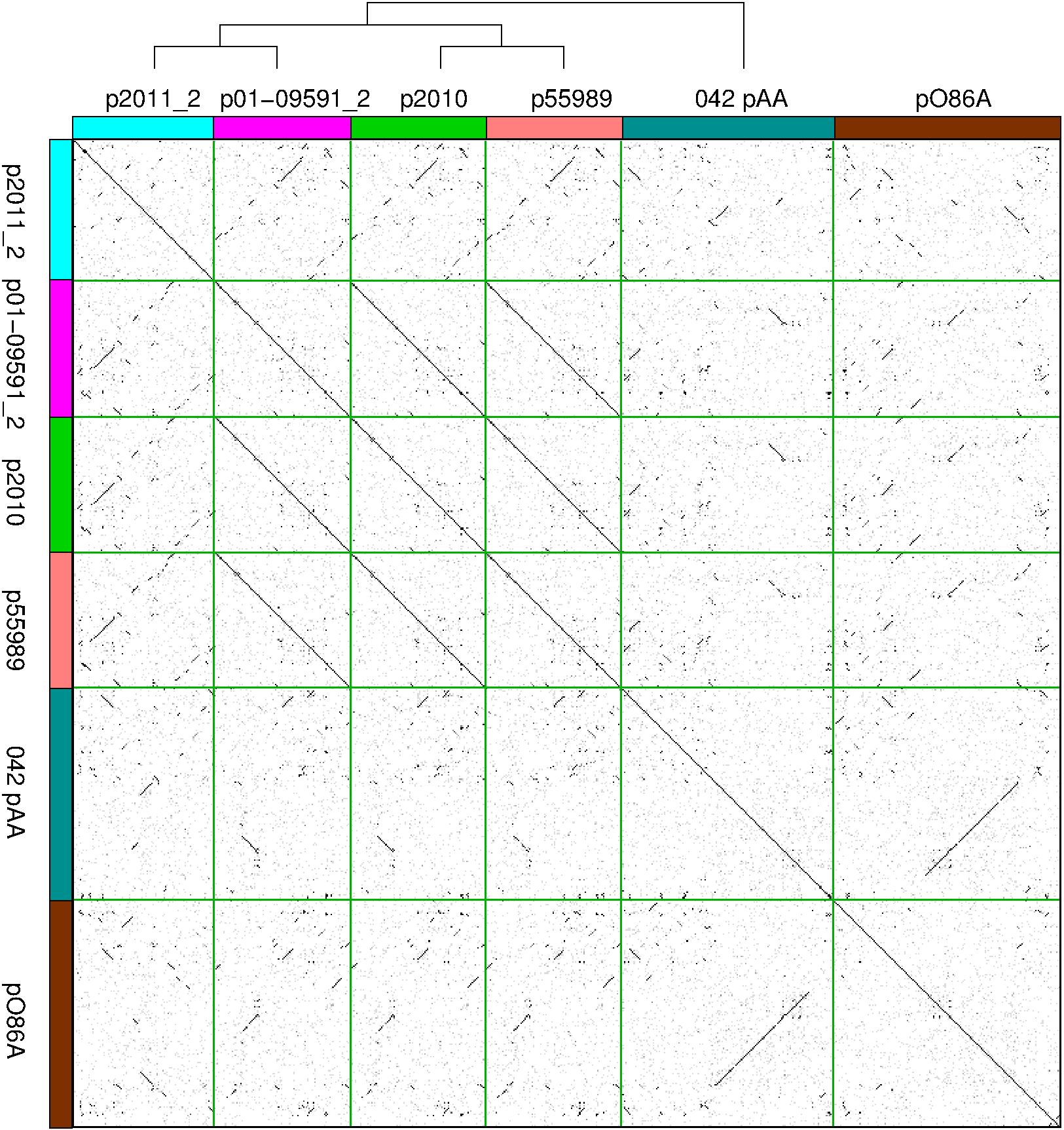

Supplement: Figure S4 — Dot plot of related pAA plasmid genomes. The likely relationship of corresponding chromosomes is shown on the top, and currently there is no complete chromosome sequence associated with pO86A. (TIF) [file pone.0033971.s004.tif]

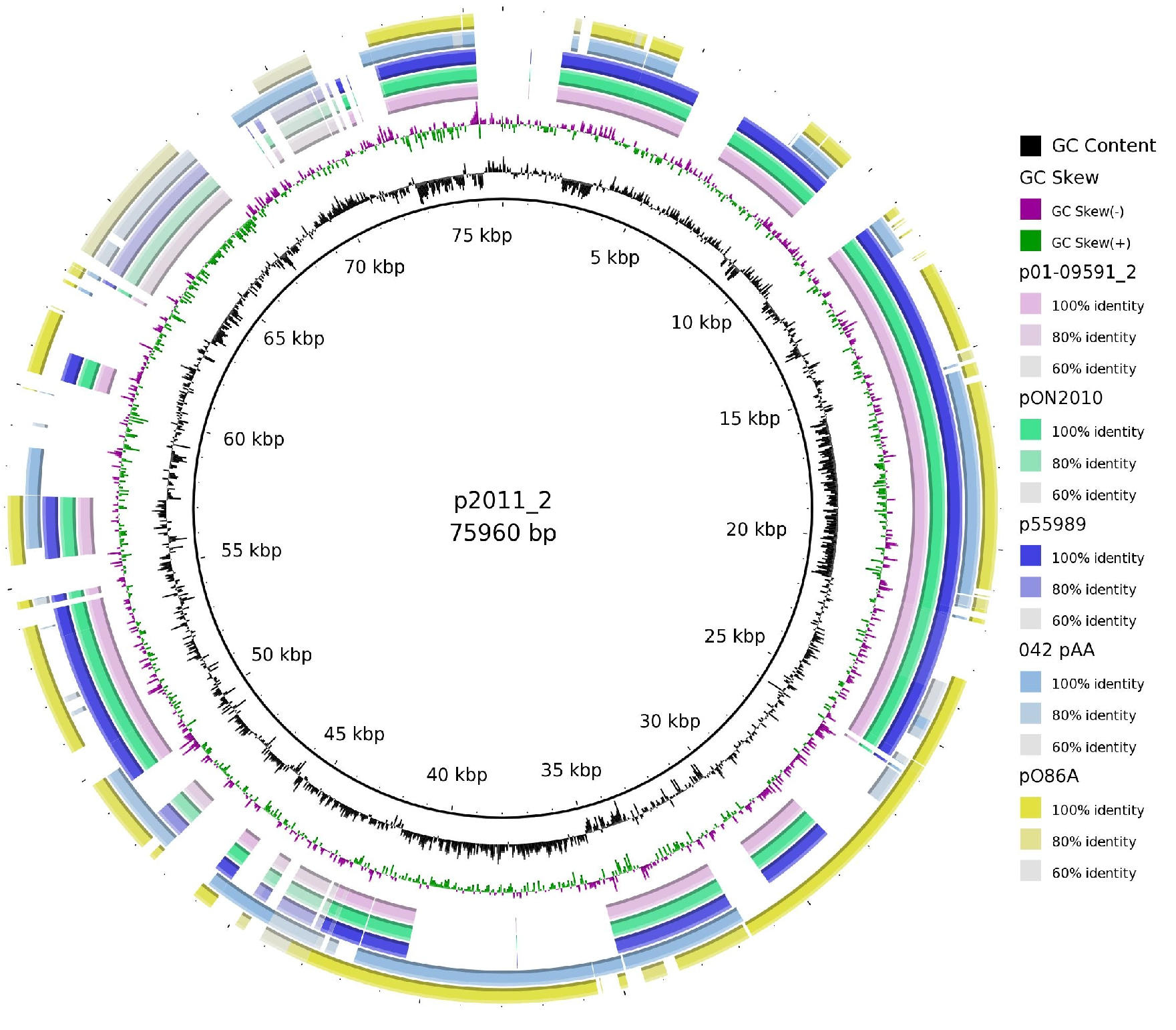

Supplement: Figure S5 — BRIG analysis of pAA plasmid genomes using the pAA plasmid in ON2011 as the query sequence. (TIF) [file pone.0033971.s005.tif]

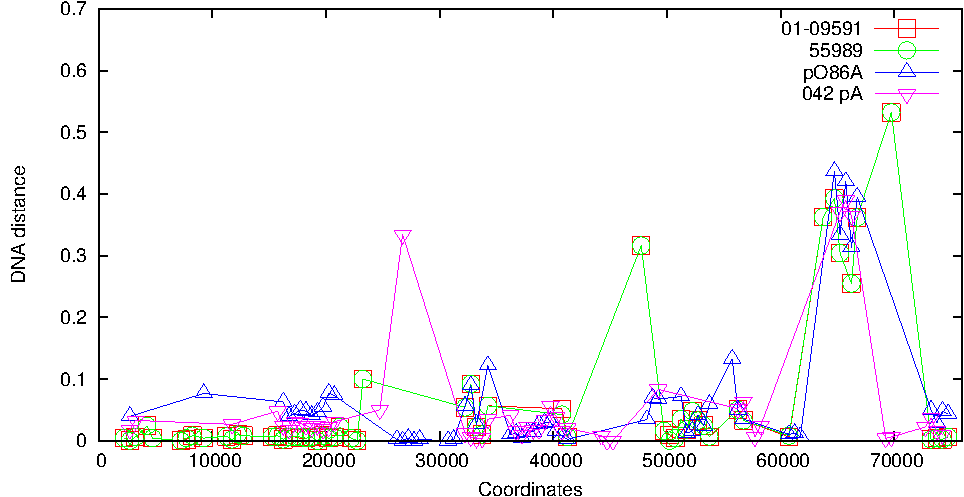

Supplement: Figure S6 — DNA distance among related plasmid genomes. Non-overlapped 500-nucleotide fragments from the second plasmid in ON2011 were used as query sequences. Each data point is based on the DNA distance between the query and subject sequence alignment. (TIF) [file pone.0033971.s006.tif]
